# NC2213: a novel methionine aminopeptidase 2 inhibitor in human colon cancer HT29 cells

**DOI:** 10.1186/1476-4598-8-65

**Published:** 2009-08-24

**Authors:** Ponniah Selvakumar, Ashakumary Lakshmikuttyamma, Umashankar Das, Hari N Pati, Jonathan R Dimmock, Rajendra K Sharma

**Affiliations:** 1 Department of Pathology and Laboratory Medicine, College of Medicine, University of Saskatchewan, Saskatoon, Saskatchewan, S7N 4H4, Canada; 2 Cancer Research Unit, Health Research Division, Saskatchewan Cancer Agency, Saskatoon, Saskatchewan, S7N 4H4, Canada; 3 Department of Biochemistry, College of Medicine, University of Saskatchewan, Saskatoon, Saskatchewan, S7N 4H4, Canada; 4 College of Pharmacy and Nutrition, University of Saskatchewan, Saskatoon, Saskatchewan, S7N 5C9, Canada

## Abstract

Methionine aminopeptidase 2 (MetAP2) is a bifunctional protein that plays a critical role in the regulation of post-translational processing and protein synthesis. MetAP2 is overexpressed in human colon cancer. In this report we screened various MetAP2 inhibitors and treated HT29 cells with various concentrations of compounds. We evaluated the expression of MetAP2 and pp60^c-src ^expressions in HT29 cells. In addition we also carried out the cell proliferation and cell cycle analysis in the MetAP2 inhibitor-treated HT29 cells. The cell cycle analysis of HT29 treated with 1.0 μM of NC2213 showed an arrest in the G2 phase followed by an induction in the percentage of cells undergoing apoptosis in the sub-G1 phase. Western blot analysis revealed that the MetAP2 expression was dose-dependently decreased when the HT29 cells were treated with the 3,5-bis(benzylidene)-4-piperidone derivative (NC2213). In addition, phosphorylation of Src, a myristoylated oncoprotein was significantly decreased by 1.0 μM of NC2213 as revealed by Western blot analysis. Furthermore, NC2213 also inhibits the expression of pp60^c-src ^in HT29 cells. Interestingly, this compound also inhibits the phosphorylation at Tyr416 of pp60^c-src ^while increasing the phosphorylation at Tyr527 of pp60^c-src^. NC2213 inhibits the growth of HT29 cells by inducing apoptosis and might be useful for the treatment of human colon cancer.

## Findings

Methionine aminopeptidases (MetAPs) are the enzymes responsible for the removal of methionine from the amino-terminus of newly synthesized proteins [1,2] which is essential for further amino terminal modifications (e.g., myristoylation of glycine by N-myristoyltransferase, NMT) [3,4]. Various reports suggested that MetAP2 plays an important role in the growth of different types of tumors [5]. Anti-sense of MetAP2 also induces apoptosis in rat hepatoma cells [6]. A recent study suggested that fumagillin effectively inhibits both liver tumor growth and metastasis in rats *in vivo *[7]. Higher MetAP2 expression was reported in malignant mesothelioma [8], malignant lymphomas [9] and esophageal squamous carcinomas [10]. The angiogenesis inhibitor TNP470, O-(chloro-acetyl-carbamoyl) fumagillol, a synthetic analogue of fumagillin, suppressed the expression of MetAP2 in human neuroblastoma and thus, MetAP2 may be an important molecular target for human neuroblastomas [11]. Earlier, we demonstrated the high expression of MetAP2 in colorectal adenocarcinoma patients [12]. It appears that higher expression of MetAP2 is required for the overexpression of NMT in colon carcinogenesis.

**Figure 1:**
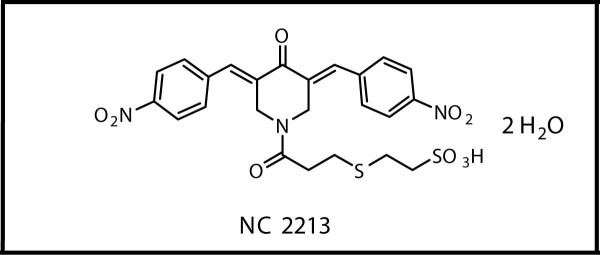
**Structure of NC2213**.

**Figure 2:**
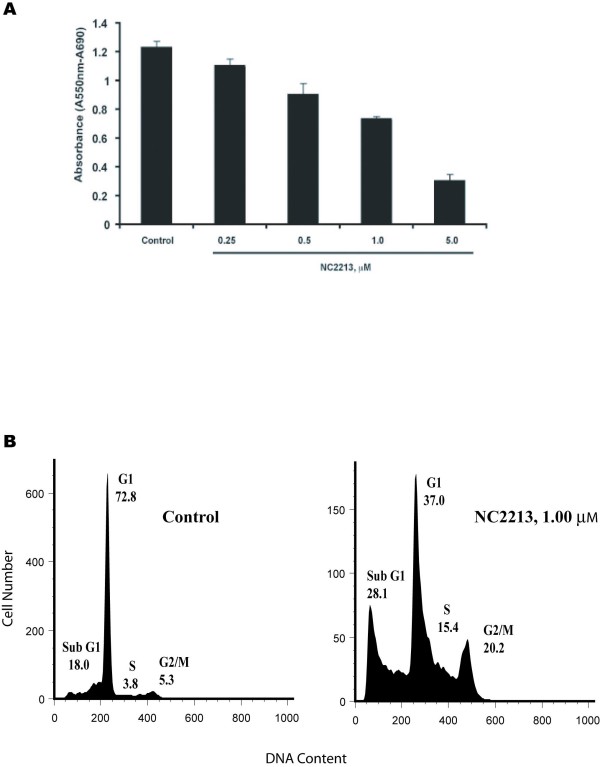
**A, cell proliferation analysis of HT29 cells**. The cells were treated with NC2213 or DMSO (control) at various concentrations for 96 hours; B, cell cycle analysis of HT29 cells. The cells were treated with NC2213 or DMSO (control) at various concentrations for 48 hours. Histograms represent the PI staining of DNA content in different phases of the cell cycle. Percentages of cells in each phase of the cell cycle are shown on the histograms. The data presented as the representative of at least three separate experiments.

The purpose of the present study is to identify a novel MetAP2 inhibitor. We screened various small molecules using a cell proliferation (MTT) assay. Among several compounds screened, we identified 2-{3-[3,5-bis[4-nitrobenzylidene]-4-oxopiperidin-1-yl]-3-oxopropylsulfanyl} ethanesulfonic acid NC2213 (Figure 1), which is structurally divergent from fumagillin, as an inhibitor of MetAP2. Various cytotoxic evaluations against human squamous cells (HSC-2, HSC-4), and leukemic cells (HL-60) displayed moderate inhibition [13]. HT29 colon cancer cells were treated with NC2213 at a concentration range of 0 to 5.0 μM for 96 hours to confirm a dose-dependent inhibitory effect. NC2213 inhibited HT29 cells, human colon cancer cell lines (HCCL), in a dose-dependent manner with an IC_50 _value of 1.2 μM (Figure 2A). HT29 cell proliferation was significantly inhibited by 1.0 μM of NC2213. The effects of NC2213 on cell cycle progression and cell death in HT29 cells were analyzed by flow cytometric analysis of the DNA content using fixed, propidium iodide-stained cells. HT29 cells were exposed to NC2213 at various concentrations for 48 hours. The addition of NC2213 (1.0 μM) seemed to abrogate the G2-M arrest and significantly increased the population of cells with sub-G (apoptotic) DNA content. The percentage of G1 cells decreased 50% with 1.0 μM NC2213 after 48 hours. The cell cycle analysis of HT29 treated with 1.0 μM of NC2213 showed an arrest in the G2 phase followed by an induction in the percentage of cells undergoing apoptosis in the sub-G1 phase (Figure 2B).

**Figure 3:**
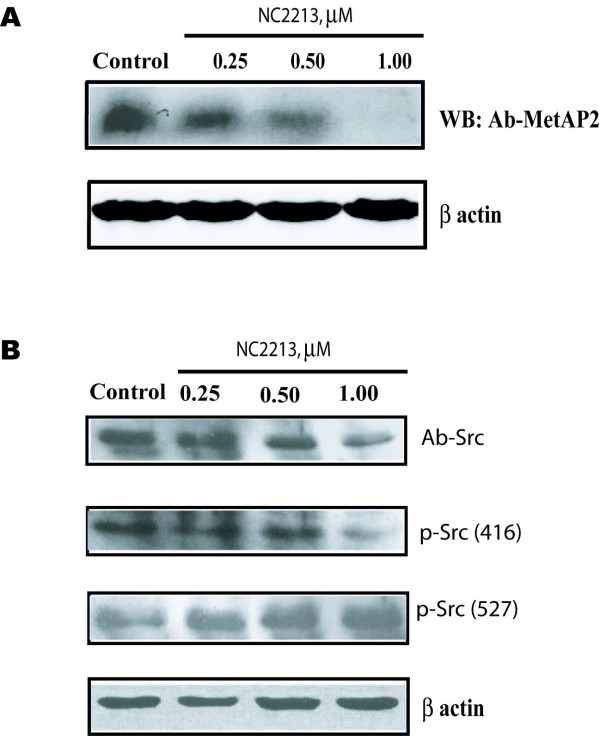
**Expression of MetAP2 and Src in HT29 cells**. A, expression of MetAP2; B, expression of Src. Cells were treated with NC2213 at various concentrations for 48 hours. Equal amounts of proteins were loaded on to each lane, subjected to SDS-PAGE, transferred to nitrocellulose membrane, and blotted with anti-MetAP2, anti-pp60^c-src^, anti-phospho-Src (Tyr527), anti-phospho-Src (Tyr416), as described under Materials and Methods. β-Actin was used as a loading control. The data presented as the representative of at least three separate experiments.

To investigate the inhibition of MetAP2 by NC2213, the dose-dependent effects of NC2213 were evaluated by Western blot analysis. HT29 cells, treated with NC2213 for 48 hours, led to a dramatic decrease in MetAP2 expression (Figure 3A). Very recently, it has been reported that MetAP2 could function as an oncogene [14]. An adenovirus transfer of cMyc increased the expression of MetAP2 in human umbilical vein endothelial cells revealed that cellular proliferation may involve a cross-talk between cMyc and MetAP2. Furthermore, various Src family tyrosine kinases, ADP ribosylation factors and eukaryotic transcription elongation factor-2 were substrates of MetAP2 which plays a significant role in the progression of metastasis [14]. From our above observations it is evident that MetAP2 may serve as a novel target for the treatment of colon cancer. MetAP2 is one such target candidate due to its inactivation by the widely investigated anticancer agent TNP470 [15-17]. A derivative of the natural product fumagillin, TNP470 has been shown to be safe and effective in the treatment of solid tumors in several animal studies and preclinical trials. TNP470 entered human clinical trials for the treatment of AIDS-related Kaposi's sarcoma, metastatic breast cancer, androgen-independent prostate cancer, pediatric solid tumors, lymphomas, acute leukemia, advanced squamous cell cancer of the cervix, and metastatic renal carcinoma [18-20]. Several MetAP2 inhibitors were studied based on the inhibition of MetAP activity [21-27]. Previously, inhibition of MetAP2 by TNP470 has been shown to activate p53 for cell-cycle arrest [25,26]. In fact, the primary mouse embryonic fibroblasts were demonstrated to be sensitive to TNP470 and other MetAP2-specific inhibitors in a p53-dependent fashion [25,26]. In contrast, the majority of transformed tumor cells are largely resistant to TNP470, likely due to the pre-existing p53 mutations in them.

c-Src is frequently observed to be activated or overexpressed in a number of human cancers, especially those of colon and breast. Activation of c-Src is achieved upon its myristoylation for proper signal transduction. Since myristoylation reaction is catalyzed by NMT, we reported that a cross-talk among the MetAP2, NMT, and N-myristoyltransferase inhibitor protein 71 (NIP71) in HT29 cells [27]. Importantly, the Src family kinases have been shown to play pivotal roles in cell-cycle progression, making them potential candidates to mediate the cell-cycle effects of MetAP inhibitors. Western blot analysis revealed that phosphorylation of Src was significantly decreased by 1.0 μM of NC2213 (Figure 3B). We further investigated the molecular events associated with NC2213-induced cell cycle arrest by measuring the phosphorylation of Src, a myristoylated protein, is elevated in human colon cancer (Figure 3B). The phosphorylation of Src at Tyr417 and Tyr527 in HT29 cells was measured by Western blot analysis (Figure 3B). These observations lead us to the possibility of developing MetAP2 specific inhibitors, which may be therapeutically useful. In addition, it is important to study the regulation of MetAP2 and its involvement of various signal transduction pathways.

The possible role of MetAP2 and NMT in cell proliferation may be due to the interaction of these enzymes with various apoptotic factors. Increased expression of NMT in p53 mutant cases suggests that wild-type p53 may have a negative regulatory effect on NMT gene expression [28]. MetAP2 plays a critical role in the proliferation of endothelial cells and certain tumor cells and thus serves as a promising target for anti-angiogenesis and anticancer drugs [10]. It has been demonstrated that there is a high expression of MetAP2 in human mesothelioma tissue, and the association of this expression with anti-apoptotic function in those neoplastic cells [8]. In addition, the inhibition of MetAP2 expression in mesothelioma cells leads to cell death and that such apoptosis is avoided in cases where there is overexpression of Bcl-2 [8]. The upregulation of Bcl-2 in colorectal cancer is well established by various investigators [28-30]. One of the other mechanisms correlating with MetAP2 and apoptosis is through caspases [8]. A recent observation suggested that mesalazine-induced apoptosis in colon cancer cells is possible through activation of caspase-3 [31]. Chen et al reported a reduction in protein levels of caspase-3, caspase-7, and caspase-9 in human colon cancer specimens [32].

In summary, we have identified a novel MetAP2 inhibitor NC2213, which is a structurally divergent to fumagillin, in HT29 cells. The MetAP2 expression was dose-dependently decreased when the HT29 cells were treated with NC2213. NC2213 also inhibits the expression of pp60^c-src ^and the phosphorylation at Tyr417 while increasing the phosphorylation at Tyr527 of pp60^c-src ^in HT29 cells. Future detailed studies related to MetAP2 and apoptosis will shed light on the involvement of this enzyme in cell proliferation.

## Materials and methods

### Materials

Anti-MetAP2 was purchased from Zymed Laboratories Inc., (USA). Anti-pp60^c-src^, anti-phospho-Src (Tyr527], and anti-phospho-Src (Tyr416] were purchased from Cell Signaling, USA. Cell culture media, anti-β-Actin and other reagents were from Invitrogen and Sigma. NC2213 was synthesized as described earlier (Figure 1) [13].

### Cell Culture and Cell Lysate Preparation

The HT29 cells were grown as described elsewhere [27].

### Cell Proliferation Assay

Cells were dissociated with 2.5 g/L trypsin and resuspended in tissue culture media. Cells (1 × 10^5 ^cells/mL) were added to 96-well plates (9000 cells/well) for 24 h at 37°C. NC2213 was added at various concentrations ranging from 0-5 μM. After the cells were incubated with NC2213 for 96 h, cell proliferation was estimated based on the cellular reduction of tetrazolium salt MTT by using a micro plate reader (BIO-RAD, Model 550 USA) at 540 nm [33].

### Cell Cycle Analysis

Cell cycle analysis was carried out as described elsewhere [34]. Cells were treated with NC2213 at various concentrations ranging from 0-5 μM. After treatment for 48 h, cells were trypsinized, washed in PBS, and fixed overnight in 70% ethanol at 4°C. At the time of harvest, the cultures were 70-90% confluent. The ethanol solution was subsequently removed after centrifugation, and cells were resuspended in a buffer containing 10 mM Tris (pH 7.5), 125 mM sucrose, 2.5 mM MgCl_2_, 0.185% NP40, 0.02 mg/ml RNase A, 0.05% sodium citrate, and 25 μg/ml PI. After incubation on ice for 1 h, cells were subjected to DNA content analysis using a FACScan cytometer (Becton Dickinson).

### SDS-Polyacrylamide Gel Electrophoresis and Western Blot Analysis

SDS-PAGE prepared according to the procedure described by Laemmli[35]. Gel transfer to nitrocellulose membrane and blocking were performed using standard procedures [36]. The blot was incubated with the primary antibodies at 1:1000, washed and probed with an anti-rabbit or anti-mouse IgG horseradish peroxidase conjugate diluted 1:2000.

### Other Methods

Protein concentration was measured by the method of Bradford [37] using bovine serum albumin as a standard.

## Abbreviations

MetAP2: Methionine aminopeptidase; NMT: N-myristoyltransferase; TNP470: O-(chloro-acetyl-carbamoyl) fumagillol; NIP71: N-myristoyltransferase inhibitor protein 71;

## Competing interests

The authors declare that they have no competing interests.

## Authors' contributions

PS and RKS conceived, and planned the experiments. AL did flow cytometry analysis. UD, HNP and JRD provided the NC2213 compound. All authors read and approved the manuscript.
